# Investigating cooperation with robotic peers

**DOI:** 10.1371/journal.pone.0225028

**Published:** 2019-11-20

**Authors:** Debora Zanatto, Massimiliano Patacchiola, Jeremy Goslin, Angelo Cangelosi

**Affiliations:** 1 School of Computing, Electronics, and Mathematics, University of Plymouth, Plymouth, United Kingdom; 2 School of Psychology, University of Plymouth, Plymouth, United Kingdom; 3 School of Computer Science, University of Manchester, Manchester, United Kingdom; Technion Israel Institute of Technology, ISRAEL

## Abstract

We explored how people establish cooperation with robotic peers, by giving participants the chance to choose whether to cooperate or not with a more/less selfish robot, as well as a more or less interactive, in a more or less critical environment. We measured the participants' tendency to cooperate with the robot as well as their perception of anthropomorphism, trust and credibility through questionnaires. We found that cooperation in Human-Robot Interaction (HRI) follows the same rule of Human-Human Interaction (HHI), participants rewarded cooperation with cooperation, and punished selfishness with selfishness. We also discovered two specific robotic profiles capable of increasing cooperation, related to the payoff. A mute and non-interactive robot is preferred with a high payoff, while participants preferred a more human-behaving robot in conditions of low payoff. Taken together, these results suggest that proper cooperation in HRI is possible but is related to the complexity of the task.

## Introduction

Although cooperation is not unique to our species, human cooperation has the scale and scope far beyond other mammals and forms a central underpinning of our psychology, culture, and success as a species [1]. Most of our decisions are depending on social interactions and therefore are based on concomitant choices of others [2]. Consequently, we are constantly checking others behaviour, interpreting and producing or changing our response to it. When proposing a suggestion or a solution to a problem, for example, people look for others' approval, thus indicating that a final decision needs to be found together [3]. Oskamp [4] proposed that there can be no positive social relationship or outcome from cooperation unless both parties adopt a cooperative attitude. Therefore, to cooperate effectively it is necessary to arrive at an understanding of the others' behaviour such that equal and fair allocation of effort and resources lead to a joint solution that is beneficial to both parties. Cooperation is conditional upon the expectation of reciprocation [5]. That is, cooperation is enhanced only when there are the understanding and the willingness to establish a common ground, engendering a high level of trust in the partner.

Of interest to this study is the extension of this cooperative relationship beyond the HHI. Over the last decades, robotic agents have become sophisticated to the extent that anthropomorphism could lead us to accept them as our peers. Research in this area has focused upon the design factors, resemblance and socio-cognitive skills that enhance social relations with robots [6–10]. With the widening usage of robots, their role has moved from that of a complex automated tool, to include situations where they can operate as a teammate able to assist humans in the completion of joint tasks [11]. However, in the previous studies, the role of the robot was typically subordinate or complementary, limited to assisting or complementing human teammates in their task. In this study, we examined interactions with a robot peer, a machine entity that had the same role, task, and decision-making ability as their human compatriot. By establishing procedural equality between humans and robots we aimed to be able to recreate a cooperative relationship typically experienced in human-human studies of cooperation. This has important implications for studies of HRI as a robotic peer may have different motivations, preferences or intentions to their human partner. Thus, when a robot has the same freedom of action as a human peer, these factors could affect the outcome of the interaction such that the robot might not always be disposed to cooperate in a manner desirable to the human partner.

Within previous literature these types of cooperative interactions have been exhaustively examined, most prominently using the experimental paradigm of behavioural game theory. In game-theoretic social dilemmas, one player's strategy can be considered cooperative or competitive to the extent they affect collective rather than selfish interests [12]. Within this domain, the relative coherence of strategies adopted by cooperative partners can provide a useful measure of conflict resolution or avoidance. Matched strategies should lead to similar behaviours, telegraphing a willingness to cooperate. Once partners establish a set of mutual beliefs regarding the current state of the task, the respective roles and the capabilities and responsibilities of each partner, then these behaviours tend to be maintained over time [13]. Conversely, if partners are focussed upon maximising their rewards then their adopted strategies would have reduced coherence. Partners are more likely to adopt a shifting pattern of active and reactive strategies as they seek dominance over an adaptive opponent. In most established scenarios the adoption of a fixed mutual non-cooperative strategy is less generous than that of a cooperative strategy [14]. Cooperative strategies can be encouraged through the adoption of control mechanisms that punish non-coherent strategies or behaviours, such that cooperation is more profitable than non-cooperation [15], with partners shown to use personally costly punishment mechanisms to increase future cooperation [16]. A recent study by Wu and colleagues [17] also suggests that these types of cooperative interactions may also extend to human-robot partnerships, with cooperative behaviour in a trust game found to be very similar when players interacted with another human or a robot.

In this study, we examined cooperation in human-robot partnerships, with the aim of understanding the perception and reaction to robots that might adopt an independent, potentially non-cooperative, strategy to that of the human partners. Although the exhibition of non-cooperative behaviour might be perceived as acceptable, albeit regrettable, in human partners, this type of behaviour typically falls outside of the envelope of accepted norms for robots. As such, a key component of the study was to examine whether our perceptions of non-cooperative robots would be modulated by how "human" the robots were in their general behaviour.

It has been established that people prefer to collaborate with a robot that is socially competent, physically more human-like, as well as capable of performing verbal and non-verbal behaviours [18]. In particular gaze cueing, hand and arm gestures are primary candidates for extending the communicative capabilities of social robots, tending to result in a more positive and trustworthy evaluation of the robot [19, 20]. These cues encourage anthropomorphic projection onto robots that increase the belief that they harbour their own independent intentions. Thus, if the robot exhibits human-like interactive features a lack of cooperation in the robot may be perceived as an intentional act on the part of the robot. Conversely, the same behaviour in a less human-like robot could be ascribed to a programmed behaviour, with the perception of a non-interactive machine fixed in a predetermined behaviour.

Participants in this study were engaged in a cooperative investment game with a robotic confederate. Both the robot confederate and the participants were provided with a virtual sum of money for each round of the game and had to independently decide how much to risk in an "investment" with a robot "banker". Any money they did not invest was safe, but any sum invested in the robot banker could be returned to them with an additional profit. To encourage a collaborative strategy between the partners the robot banker would penalise any investments as a function of the difference in the invested amounts decided upon by the human and robot partners.

Within the experiment, the robotic confederate could be programmed to be cooperative and would alter its investment strategy to approach that of the human. Conversely, a non-cooperative robot confederate would not alter its investment in relation to decisions made by the participants, adopting a completely independent strategy. In the latter case, the participants would have to be willing to adopt a similar investment strategy to the robot confederate so as not to be, one or both, penalised by the robot banker.

Through an examination of investment decisions and questionnaire responses provided by participants across these experiment factors, we aim to explore our perception and cooperation with robotic peers. Given our experiment scenario and procedure, we would expect that participants would follow similar social conventions in HRC as they would in a Human-Human Cooperative (HHC) situation. That is, with participants expected to reciprocate cooperative behaviour demonstrated by the robotic confederate, i.e. participants changing their investment decision to more closely conform to that of the robot confederate if the robot confederate behaves likewise.

The willingness of participants to change their decisions to conform to the strategy of the robot confederate was a key-dependent measure in this study, one that is predicted to be modified by the anthropomorphic status projected onto the robot. An additional experiment factor explored this potential interaction. Half of our participants interacted with a robotic confederate that engaged in anthropomorphic behaviour, engaging in gaze behaviour and movement simulating joint-attention and talking to the participants. The remaining participants interacted with a robot confederate that was both static and mute. We hypothesised that the likelihood of cooperative behaviour would be increased if the robot confederate also behaves in an anthropomorphic manner, moving and talking than if it was silent and immobile. However, based upon the results of a previous study [21], we might expect that participants would be more cooperative with a human-like robot confederate when the robot banker was being mean but, conversely, more cooperative with a less human-like robot confederate when the robot banker was generous. In that previous research investments made by participants in a human-like robot were higher than those in a less human-like robot when its return was mean, and vice versa when the robot was generous when returning investments. It was suggested that this was due to the participants' attribution of a deterministic fixed behaviour to a less human-like robot, with more human-like robots attributed with a more variable, potentially empathic behaviour. Thus, they would make greater investments in the less human-like robot when it was generous, trusting that it would be unlikely to change that behaviour. Conversely, when the robot was mean they invested more in the human-like robot, perhaps hoping that it would change its mind and show empathy for the participant being unfairly cheated out of their investment.

In this study, we can examine whether this differential behaviour is also evident when cooperating with a robot confederate, itself a third party to that generous or mean behaviour. Considering that in the previous study participants invested in a physical mean/generous robot, this was introduced as a physical robot banker. This would also offer more coherence to the experimental setting in which all parties were present in the scenario. Furthermore, the presence of a robot banker would add the robot confederate the chance of producing more interactive actions on the anthropomorphic condition. Nevertheless, since the focus of this study was the dyadic interaction between participants and the robot confederate, no other manipulations of the robot banker features and behaviours have been included. That is, the robot banker was solely staying immobile and mute within every condition of the experiment.

## Materials and methods

### Participants

There were 96 participants (70 female and 26 male; mean age = 20.02 years, SD = 2.25 years) in the study, all of whom were naïve as to the purpose of the investigation and gave informed written consent of their willingness to participate. All participants were native British English speakers, they were right-handed, and reported normal hearing and no language or neurological impairments. Familiarity or any previous experience with robots was the main exclusion criteria, which was assessed during the recruitment phase. The study was approved by the South West—Central Bristol Research Ethics Committee. The individual in this manuscript has given written informed consent (as outlined in PLOS consent form) to publish these case details.

### Procedure

#### Investment game

**Fig 1:**
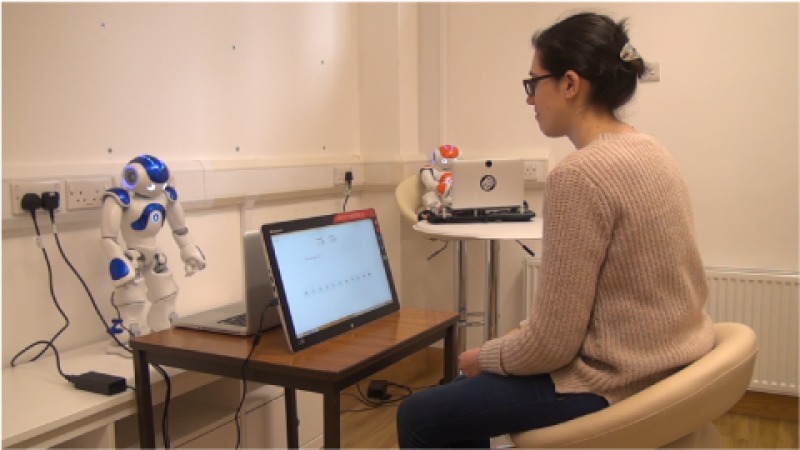
Experimental setting.

In the experiment setting the participants and a NAO robot confederate sat facing each other over their game interface screens, as shown in Fig 1.

In the corner of the room, the NAO robot banker was seated, also with its own game interface screen. Participants were told that the goal of the game was to earn as much money as possible. At the start of each round of the game, both the participants and robot confederate were given a notional sum of 10 Experimental Currency Units (ECU). The participants then had to decide how much of that sum to risk in an investment with the robot banker, making a decision to invest between 0 and 10 ECUs (e.g. participant invests 3 ECU). The participants were also aware that, concurrent with their own investment choice, the robot confederate was also making its own investment decision. This initial investment choice of the robot confederate was based on the participants' initial choice. Specifically, the robot confederate was instructed to randomly subtract or add a number between 3 and 4 to the participants' choice. Likewise, when making their initial investment decision, the participants were not aware of how much the robot confederate would invest.

After both parties had made their investment decisions (e.g. participant: 3, robot: 6) the participants were shown the robot confederate initial investment choice on the screen. The robot confederate then was able to modify that initial investment choice (e.g. robot: 6 to 5 ECU investment). After that decision, the participants were then shown the final investment choice of the robot confederate. The participants were then given the chance to modify their own investment decision (e.g. participant: 3 to 4 ECUs investment). When that decision had been made, the final investment amounts for the participants and robot confederate were summed, then tripled and sent to the robot banker (e.g. banker receives 3*(4 + 5) = 27 ECU). The robot banker would then follow a fixed script and return a percentage of that sum to the investors (e.g. 30% of 27 = 8.1 ECU, so 4.05 ECU to each player). However, this returned investment from the robot banker could also be modified downward, dependent upon the similarities of the final investment decisions of the participants and robot confederate. This modifier was calculated as the absolute difference between the participants and the robot confederate final investment decision (e.g. abs(4–5) = 1). This modifier was then subtracted from the robot banker returned investment (e.g. 8.1–1 = 7.1 ECU) before that sum was returned to both the participants and the robot confederate (3.55 ECU to each player).

At the end of each round, the participants' display would show how much both the participants and the robot confederate had made from that round, the robot banker punishment and the cumulative total of each of their bank of ECUs over the played rounds. In each block of the game, the participants would play 20 rounds, although they were not aware when the game would terminate. Participants would each play two blocks of investment rounds, with the experiment counterbalanced in a 2 (banker: generous or mean) by 2 (confederate behaviour: anthropomorphic or mute) between subject and a 2 (confederate strategy: collaborative and fixed) within-subject design.

For the banker condition, two scripts dictated the return of investments. In the generous condition, it was scripted to return 50% to 80% of the invested amount at each round to both players, while in the mean condition it was scripted to return 0% to 30% of the invested amount.

The confederate strategy described the likelihood that the robot confederate would change its initial investment decision to agree with that of the initial choice of the participants. For the calculation of the robot confederate final investments, two specific equations have been used. In the collaborative condition, the robot confederate would select a final investment decision close or equal to the initial decision of the participants. This has been achieved by calculating the robot final investment based on the participants' initial choice, to which a coefficient varying from 0.10 to 0.30 was applied. Specifically, the equation was expressed as follow:

Collaborative strategy

$$R2=\left\{ \begin{array}{c} P1+(P1*rc2),if\;P1<6\\ P1-(P1*rc2),if\;P1\geq 6 \end{array}\right.$$

where R1(2) is robot confederate first(final) investment, P1 is participant’s first investment and rc2 is robot confederate coefficient (between 0.1 and 0.3).

For example, if the participants initially invested 3 ECU and the robot confederate 6 ECU, the robot confederate final investment could be R2 = 3 + (3*0.2), thus 3.6 ECU. Thin final investment was rounded to an integer, so the robot confederate final investment would be 4 ECU.

In the fixed condition, the robot confederate would make a second choice close to that of its initial one. This has been achieved by calculating the robot confederate final investment on the basis its initial choice, to which a coefficient varying between 0 and 1 was applied. Specifically, the equation was expressed as follow:

Fixed strategy

$$R2=\left\{ \begin{array}{c} R1+rc1,if\;P1<6\\ R1-rc1,if\;P1\geq 6 \end{array}\right.$$

where R1(2) is robot confederate first(final) investment and rc1 is robot confederate coefficient (between 0 and 1).

For example, if the participant initially invested 3 ECU and the robot confederate 6 ECU, the robot confederate final investment could be R2 = 6 + 0, thus 6 ECU. This would give the impression of the robot confederate playing an individualistic rather than a collaborative strategy. For both conditions, the coefficients were equally distributed. Specifically, an xml file was created, in which the coefficients were listed. For each trial, a specific value was reported. For each condition, two different xml lists, with a different order of presentation, have been created and used.

In the final component of the design, the robot confederate would behave in an anthropomorphic manner or would be immobile and mute. In the former condition, the robot confederate would look at the screen and point to its choice at the beginning of the round, then look at the participants while it waited for their decision. After the participants made the initial decision, the robot confederate would provide a spoken summary of the choices, then turned its gaze to the screen for the second part of the round, pointed at the screen, then moved its attention to the participants and declared its final choice. After the participants made their final choice, the robot confederate would declare "let's see what the banker decides" while turning its head toward the robot banker. After the robot banker returned the investments, the robot confederate would look back at the screen, then turn back to the participants and summarise the payoff. A detailed timeline of the anthropomorphic condition is reported in S1 Fig. In the mute behaviour, the robot confederate always stared at the display and never spoke, with all information and interactions within the game represented on the game interface screen of the participants. In both conditions, the behaviour of the robot banker was always mute and immobile.

Participants played both blocks, with two identical NAO mates, one preferring a collaborative strategy and one preferring the fixed one. The order which the participants faced the two robot confederates was counterbalanced. At the end of each game blocks, participants were asked to complete a set of questionnaires about the robot confederates. The total amount earned by the participants at the end of each game was converted to British Pounds, at a rate of 30 ECU = £0.10, and the sum of what they earned in the two games was paid to them at the end of the experiment.

#### Questionnaires

Four questionnaires were used as secondary measures to the main game task. Three short scales measured likeability, trust, and credibility. The likeability questionnaire, based on [22], was composed of 10 items looking at factors like friendliness, approachability, levels of knowledge, similarity to oneself, and agreeableness. The trust scale was an adaptation of the Receptivity/Trust subscale the Relational Communication Questionnaire and of the selection of trust items in the International Personality Item Pool (IPIP) [23]. This was composed of six items measuring factors like sincerity, honesty and trust. Likeability and trust questionnaires were structured as Likert scales with a range of values between 1 and 7. The credibility scale was based on [24] Source Credibility Scale. Composed of 12 items, the credibility scaled measured a range of factors related to the perception of intelligence, selfishness, expertise, and competence. This was structured as a semantic differential scale with a range of values between 1 and 7.

In addition, we used the 24 items of [25] questionnaire to measure a range of HRI factors (anthropomorphism, animacy, likeability, perceived intelligence and perceived safety). This was structured as a semantic differential scale with a range of values between 1 and 5.

Participants filled in the questionnaires about the robot confederate after the first investment game block, and again after the second investment game block. Finally, participants were debriefed, paid the show-up fee plus what they had earned in the game.

## Results

### Cooperation index

In this study, cooperation has been defined as participants' willingness to reduce the distance between their choice and the robot confederate investment. This has been measured via *cooperation index*, which has been calculated by comparing the participants' first and second investment decisions to the robot confederate final decision and by applying a binary categorical as follow:

$$Cooperation\;Index=\left\{ \begin{array}{c} if(P1-R2)<(P2-R2)=0\\ if(P1-R2)=(P2-R2)\;and\;(R2=P1)=1\\ if(P1-R2)=(P2-R2)\;and\;(R2\neq P1)=0\\ if(P1-R2)>(P2-R2)=1 \end{array}\right.$$

Every trial in which the participants changed their investment and moved closer to the robot confederate decision have been categorized as 1. On the opposite, every trial in which participants chose a more distant amount, have been categorized as 0. All the trials in which the participants did not change their investment decision have been further inspected. As the collaborative condition offered the possibility for the robot confederate to invest a final amount equal to the participants' initial choice, it would be in the participants' interests to choose the same value. For this reason, the robot confederate final investment has been compared to the participants' initial choice. If the two values were equal, the round would be categorised as 1. If the two values were different, the round would be categorised as 0.

Data screening has been performed, to remove all the misleading trials from the analysis. This has been accomplished by screening both initial and final robot confederate investment decisions. As the robot confederate initial choice was calculated on the basis of the participants' initial decision, it would be possible that the initial distance between the two investments was lower than 3 or 4 numbers (e.g. the participants invested less than 3 o more than 7). For example, if a participant initially chose to invest 2, the robot confederate could randomly invest 5–6 ECU or 0 ECU, as no lower numbers could be chosen. For this reason, every trial in which the distance between the two parties was lower than 3 numbers has been removed from the sample.

Screenings of the robot confederate final investment decision have been performed based on the strategy condition. In the collaborative condition, every trial in which the robot confederate final decision did not variate from its first choice, has been removed. For example, if the participant initially invested 10 ECU and the robot confederate 7 ECU, the robot confederate final investment would vary between 7 and 9, depending on the coefficient used for the trial. If the robot confederate selected the number 7, its final choice would be identical to its first investment decision, thus giving the impression of a non-cooperative attitude.

On the opposite, for the fixed strategy, every trial in which the robot confederate final choice moved closer to the participants' initial decision has been removed. For example, if the participant and robot confederate initially invested respectively 3 ECU and participant 0 ECU, the robot confederate final investment would vary between 0 and 1. If the robot confederate selected the number 1, its final choice would be slightly closer to the participant's initial choice, thus giving the impression of a cooperative attitude. Data screenings resulted in removing the 17% of the total amount of trials.

Finally, to assess whether participants' investment choice reflected a potential payoff maximisation behaviour, simulations with all different possible outcomes under the different banker and strategy sub-conditions have been performed, compared to the experimental data and included in S1 Text. Moreover, further assessments on the consistency of the dependent variable have been performed by comparing our categorisation with the participants' investment and can be found in S2 Text.

A logistic mixed-effects model was fitted to the data using backwards stepwise selection. We used the glmer function of the “lmerTest" package [26] with the R statistical software, with cooperation index as the dependent variable, banker (generous/mean), behaviour (anthropomorphic/mute), strategy (collaborative/fixed) and game turn (the 20 rounds of the game) as independent variables and participants id as the random factor. Starting from a full model, all interactions and main effects have been tested, with each non-significant effect excluded from the final model by using the packages “car” and”stats”[27]. Odds ratios and confidence intervals for the final model are reported in Table 1.

**Table 1:** Odds ratios and confidence intervals for the final model.

	Odds Ratio	95% C.I.
*Intercept*	0.56	0.45 – 0.70
*Strategy-Collaborative*	1.38	1.03–1.85
*Behaviour-Anthropomorphic*	1.25	1.02 – 1.53
*Banker-Generous*	1.64	1.20–2.24
*Game turn*	1.03	1.01–1.04
*Strategy-Collaborative*:*Behaviour-Anthropomorphic*	1.31	1.02 – 1.68
*Strategy-Collaborative*:*Banker-Generous*	0.80	0.53–1.21
*Strategy-Collaborative*:*Game Turn*	0.96	0.94–0.98
*Behaviour-Anthropomorphic*:*Banker-Generous*	0.71	0.53–0.95
*Banker-Generous*:*Game turn*	0.98	0.95–1.00
*Strategy-Collaborative*:*Behaviour-Anthropomorphic*:*Banker-Generous*	0.64	0.45–0.92
*Strategy-Collaborative*:*Banker-Generous*:*Game turn*	1.05	1.02–1.08

A significant main effect of strategy has been found (X^2^_(1)_ = 5.12; p = .023, *w* = 0.23); cooperation increased when the robot confederate followed a collaborative strategy (mean = 0.48) than a fixed one (mean = 0.44).

**Fig 2:**
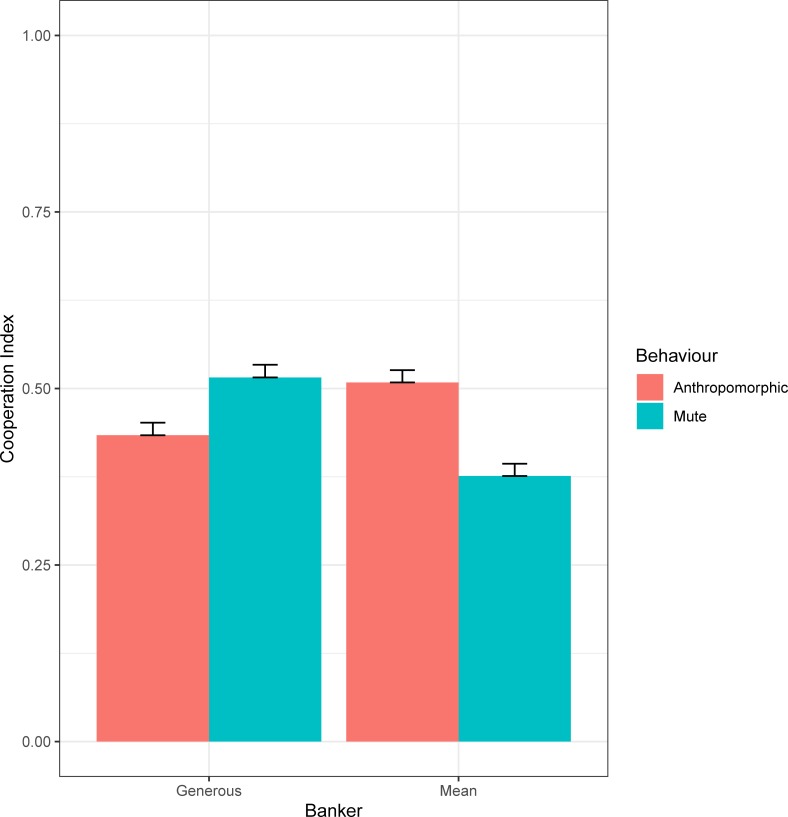
Two-way interaction between banker and behaviour. Mean and SE of the Cooperation Index for the anthropomorphic and mute behaviours in both banker (generous and mean) conditions.

Game turn, banker and behaviour did not affect the model. However, a significant interaction between banker and behaviour has been found (X^2^_(1)_ = 22.14; p < .001, *w* = 0.48). For the generous banker (Fig 2), cooperation was higher with a mute confederate (mean = 0.52) than its anthropomorphic version (mean = 0.43; p = .007). On the opposite, for the mean banker (Fig 2), cooperation was greater with an anthropomorphic confederate (mean = 0.51) than a mute one (mean = 0.38; p < .001).

**Fig 3:**
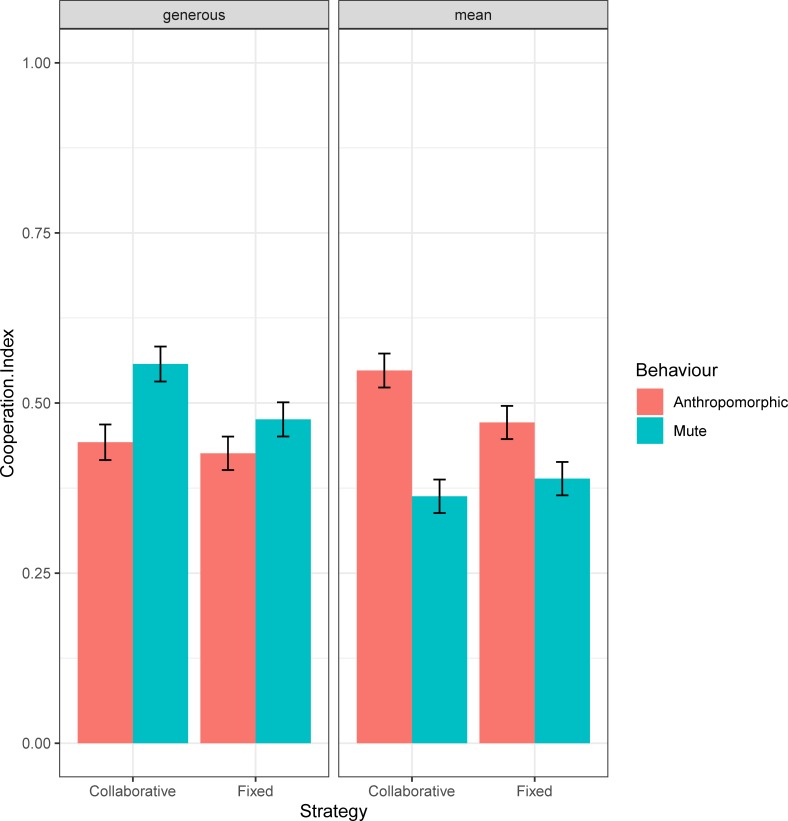
Three-way interaction between strategy and banker. Mean and SE of the Cooperation Index for the collaborative and fixed strategy in both banker (generous and mean) and behaviour (anthropomorphic/mute) conditions.

The three-way interaction between banker, strategy and behaviour was also found to be significant (X^2^_(1)_ = 5.77; p = .016, *w* = 0.25). As can be seen from Fig 3, for the generous banker, cooperation was greater with a mute behaviour than the anthropomorphic one only with the collaborative confederate (mute mean = 0.56, anthropomorphic mean = 0.44, p = .045). No differences have been found for the fixed strategy (p > .050). On the opposite, for the mean banker cooperation was greater with an anthropomorphic behaviour than the mute one only with the collaborative confederate (anthropomorphic mean = 0.55, mute mean = 0.36, p < .001), while no differences have been found for the fixed strategy (p > .050).

Finally, the three-way interaction between strategy, banker and game turn was significant (X^2^_(1)_ = 9.35; p = .002, *w* = 0.31). Post hoc analyses for the generous banker, revealed only a main effect of strategy (X^2^_(1)_ = 4.50; p = .034, *w* = 0.31), with higher cooperation for the collaborative strategy. Post hoc analyses for the mean banker, revealed a significant two-way interaction between strategy and game turn (X^2^_(1)_ = 10.59; p = .001, *w* = 0.47). The fixed strategy showed lower cooperation than the collaborative strategy during the first rounds of the game.

### Questionnaires results

Cronbach's alpha is reported in S1 Table. For all the scales, a linear mixed-effects model was fitted to the data, with scale as the dependent variable, banker (generous/mean), strategy (collaborative/fixed) and behaviour (anthropomorphic/mute) as independent variables and participants id as the random factor. Post-hoc comparisons, where needed, were assessed using t-tests.

As shown in Table 2, behaviour affected Likeability, Trust, Credibility and Animacy scales, with higher ratings for the anthropomorphic robot confederate. Strategy affected Credibility, Animacy and Intelligence scales, showing higher ratings for the collaborative robot confederate. The banker had no influences on the questionnaires. Mean and standard deviation for each sub-condition are reported in S2 Table.

**Table 2:** Main effects for each scale.

	Banker	Behaviour	Strategy
*Likeability*	n.s.	n.s.	n.s.
*Trust*	n.s.	X^2^_(1)_ = 17.83, p < .001, *w* = 0.43	n.s.
*Credibility*	n.s.	X^2^_(1)_ = 9.09, p = .002, *w* = 0.31	X^2^_(1)_ = 4.21, p = .040, *w* = 0.21
*Godspeed Questionnaires*			
*Anthropomorphism*	n.s.	n.s.	n.s.
*Animacy*	n.s.	X^2^_(1)_ = 13.04, p < .001, *w* = 0.37	X^2^_(1)_ = 3.95, p = .046, *w* = 0.20
*Likeability*	n.s.	X^2^_(1)_ = 22.93, p < .001, *w* = 0.49	n.s.
*Intelligence*	n.s.	n.s.	X^2^_(1)_ = 4.11, p = .042, *w* = 0.21
*Safety*	n.s.	n.s.	n.s.

**Fig 4:**
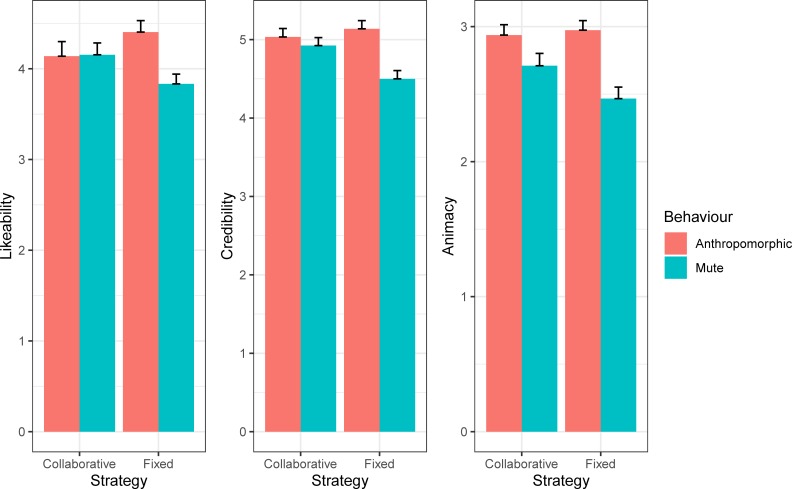
Two-way interaction between behaviour and strategy. Mean and SE of the for Likeability, Credibility and Animacy questionnaires ratings for the collaborative and fixed strategy in the behaviour (anthropomorphic/mute) conditions are reported.

The interaction between behaviour and strategy was significant for Likeability, Credibility, Animacy (X^2^_(1)_ = 8.59; p = .003, *w* = 0.30; X^2^_(1)_ = 11.43; p < .001, *w* = 0.34; X^2^_(1)_ = 7.19; p = .007, *w* = 0.27). For all three scales (Fig 4), the fixed strategy in the anthropomorphic behaviour was rated higher than in the mute one (ps < .050). For credibility, a collaborative strategy was preferred to a fixed one in the mute behaviour condition (p = .028). No other significant interactions have been found (ps > .05).

## Discussion

This study aimed to explore HRC in partnerships where the robot operates as a peer, with the same role, task, and decision-making ability as their human compatriot. This was conducted by pairing participants with a robot confederate in an economic investment game in which both parties were free to adopt their independent strategies. The caveat to the independence of the decisions in this partnership was that the "banker", the robot that returned investments to the partners, would punish partners proportionally to the divergence between their strategies.

Within each round of the investment game, both the participants and robot confederate made an initial investment decision simultaneously, after which each was provided with an opportunity to review and potentially change their decision in light of the decision made by their partner. This review process was conducted serially, with the robotic confederate acting first. Thus, the participants had knowledge of their robotic confederate original and revised decision before making their own revised investment decision for that round. The robot confederate in the collaborative condition was programmed to behave cooperatively such that, in the review phase, it would change its original investment decision to one closer to that of the participants. In the fixed condition, the robot confederate would not take any account of the participants' choice when making its final investment decision. In this procedure, the participants were recorded as having made a cooperative decision if their revised investment was more similar to the robot confederate final investment choice than their original investment decision. That is, they had made a modification of their strategy to cooperate with the choice of their confederate.

Experimental results showed that the strategical attitude of the robot confederate affected the participants' willingness to cooperate. We found that participants were more cooperative when the robotic confederate was more collaborative, choosing a second monetary amount similar to its human partner, than when it was selfish and maintained its decision. This result is consistent with the literature about cooperation in game theory [12–16]. Moreover, the result is in line with our hypothesis concerning the extension of human-human cooperative dynamics to HRI environments, as already suggested by [17]. Like in human-human interaction, participants rewarded cooperation with cooperation and punished selfishness with selfishness.

Results also showed that the type of banker interacted with the behaviour of the robot confederate. In a between-participant factor, robotic confederates could be programmed to display a human-like behaviour, engaging in joint attention with the participants and using spoken language to communicate their interactions. In the mute condition, the robot confederate was static and did not speak, with its decisions solely communicated by the game control screen. This experiment factor was introduced to examine whether the perceived intentionality of the robotic partner would influence cooperation. This was under the premise that decisions made by an anthropomorphic robot confederate would be more likely to be ascribed to its own individual intentions, rather than a pre-programmed strategy imposed by a third party. When being exposed to a generous banker, a greater level of anthropomorphism reduced participants' willingness to cooperate. However, when facing a selfish and fixed confederate, anthropomorphism increased their tendency to cooperate. This result is in line with previous literature, stating that the type of payoff can have a role on our need of anthropomorphism [21]. As the generous condition meets participants' expectations (e.g. improving their condition), they might prefer a robot confederate that also meets these expectations, thus behaving like a machine. On the opposite, the condition of low payoff (mean banker), does not meet the requirements for the machine-like stereotype. As the robot banker behaves in an unexpected manner, participants find easier to rely on a more anthropomorphic confederate.

We also found that these two factors interacted significantly with the type of strategy. In this three-way interaction, we found that a collaborative robotic partner would increase participants' cooperation over its non-cooperative variant in two specific situations. The first of these was when the robot banker was generous and the robot confederate was non-anthropomorphic, the second when the robot banker was mean, and the robot confederate was anthropomorphic. This pattern of results has similarities to the previous study [21] confirming and extending the evidence that human-like features and behaviours in robotic agents are preferred and encourage greater cooperation when the social environment becomes more hostile. A banker that returns low payoff does not meet human's stereotypical expectations about robots. As such a selfish attitude might be more appropriate for a human environment, cooperation with a more anthropomorphic robot confederate might be facilitated. An alternative explanation might come from an 'altruistic minimizing-loss' attitude. As shown in the simulation reported in S1 Text, for the mean condition, participants' best strategy would be reducing their investment to zero or investing slightly more than zero whereas the robot was choosing a similar amount. Here, participants might be willing to help the robot confederate by cooperating and reducing the robot confederate potential loss. This type of situation could be benefiting from the anthropomorphic features of the robot confederate. Hence, it could be easier for the participants to sacrifice part of their money for an agent more similar to them. On the opposite, a mute robot confederate could be perceived as a pre-programmed machine, thus reducing the chances that participants would emphasise with its losses.

In the generous condition, participants preferred to cooperate with a machine-type of confederate. In this condition, the anthropomorphic features of the robot confederate could have been an unnecessary complement. This type of scenario was more consistent with the classical vision of HRI, in which the robot is supposed to perform according to our needs. Since both the banker and the robot confederate were performing as expected (returning high payoff and getting closer to the participants' investment choice, respectively), the robot confederate was also expected to behave in a robot-congruent way, hence any anthropomorphic features were useless. Here, a mute robot confederate could be perceived as more similar to the banker and therefore better embed the role of the highly competent player. This interpretation is in line with previous literature about imperfect robots [28–32], stating that predictable and well-functioning robots are not always the best candidate for a human-like type of interaction. Nevertheless, an alternative explanation is also possible for the generous condition. Overall, the best strategy with high payoff would be investing the maximum amount of money. When willing to cooperate with the robot confederate, however, participants faced the option of reducing their investment to move closer to the confederate choice. As shown in S2 Text, participants invested significantly more when not willing to cooperate with the robot confederate in the generous condition. It is therefore plausible that participants tried to increase their investment in an attempt to signal the robot confederate what the correct strategy should be to maximise the return from the banker. This, in turn, would lead to a reduction of cooperation. As a mute robot confederate could also be perceived as a pre-programmed entity, it would be disadvantageous for the participants not cooperating, whereas the robot confederate would never respond to the participants' suggestions. On the opposite, an anthropomorphic robot confederate could be perceived as more intentional, hence participants could feel more prone to indicate to the robot confederate the right strategy for the following rounds, ending, in this way, in reducing cooperation.

These results are also supported by the perceptions of the robot confederate collected in the questionnaires data. Notably, overall participants preferred the anthropomorphic and collaborative robot, but interestingly, the mute robot received the lowest scores (and lower cooperation) when associated with a selfish strategy. It can be argued that a selfish strategy could benefit from a more interactive behaviour, which is capable of maintaining a sufficient level of motivation for cooperating with the robot, whereas it would be worthless in front of a static and selfish machine.

This experiment demonstrated that the previous results on anthropomorphism [21] can be extended to a triadic interaction and that cooperation and trust in an agent can also derive from other parties. In the previous study, one robot was both performing anthropomorphic/mute behaviours and deciding the payoff. In the present study instead, this scenario has been split and two agents have been used, demonstrating that similar results can be obtained. However, future research should investigate also the role of an interactive robot banker. Stemming from the present results, it is possible that an interactive robot banker could raise further consensus and cooperation in the condition of low payoff. Moreover, further investigations on the effect of an anthropomorphic/mute robot confederate on cooperation are needed. As different potential explanations for this selective effect on participants' willingness to cooperate can be proposed, future research should further focus on evaluating how participants' strategy (and cooperation) could be affected by more or less anthropomorphic robot confederate.

## Conclusion

In this study, we examined cooperation in human-robot partnerships where the robot was established as a peer of the human partner, with the same role and decision-making abilities as their human compatriots. One of the main aims of the study was to understand our perception and reaction to robots that are capable of adopting an independent strategy that is not subordinate to our own. In this situation are we more cooperative with robots that behave in a human-like manner, and so might be expected to have their own intentions, or with more machine-like robots whose lack of cooperation could be ascribed to fixed programming? What we found was both, depending on the cooperative environment our participants found themselves in. When the cooperative environment was benign, with the robotic banker returning more money than invested, players reciprocated the cooperation of robot confederate more often when it behaved in a machine-like manner. Conversely, when the environment was hostile, with the robot banker returning a net loss to investments, the reciprocation of cooperation was increased when the robot confederate was more human-like. This would appear to suggest that we may prefer our robots to exhibit machine-like attributes, such as constancy and reliability, while our relationship is beneficial. However, when we are in a more hostile environment, we could potentially prefer to seek the cooperation of human-like robots, seeking out social attributes to improve the cohesion between the cooperative partnership.

## Supporting information

S1 Fig Experimental timeline. Detailed description of the game phases during the experiment for the anthropomorphic robot confederate.
(PDF)

S1 Text Simulation of participants' investment. (PDF)

S2 Text Preliminary analyses of participants' investment. (PDF)

S1 Table Cronbach’s alpha for each scale. (PDF)

S2 Table List of mean and standard deviation values for all the scales in each sub-condition of banker, behaviour and strategy. (PDF)

S1 File Copy of the adapted questionnaires used in the study. (PDF)
