# Supplementary material for: Investigating cooperation with robotic peers
Source: PLoS One. 2019 Nov 20;14(11):e0225028. doi: 10.1371/journal.pone.0225028 (PMC6867652; doi:10.1371/journal.pone.0225028)

S1 Fig. Experimental Timeline.

Detailed description of the game phases during the experiment for the anthropomorphic robot confederate

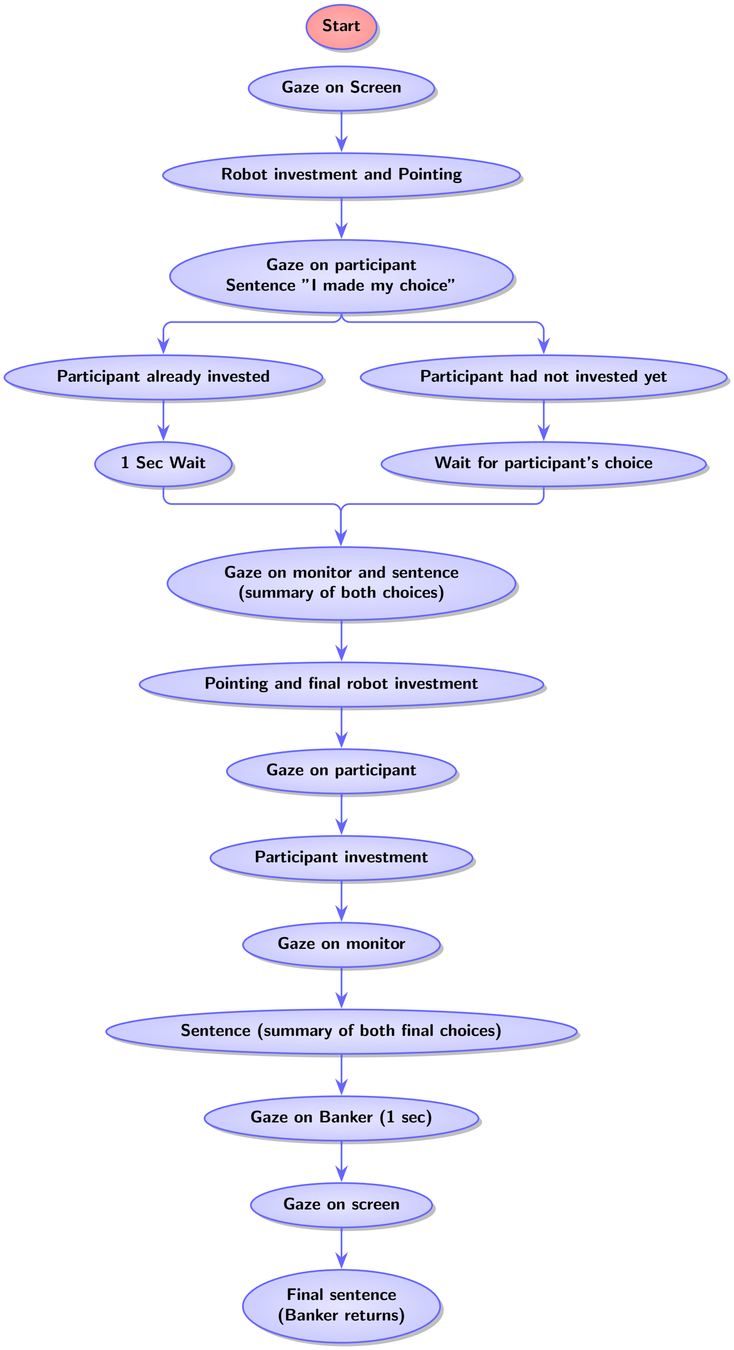

Supplement: S1 Fig — Detailed description of the game phases during the experiment for the anthropomorphic robot confederate. (PDF) [file pone.0225028.s001.pdf]
