# Supplementary material for: Investigating cooperation with robotic peers
Source: PLoS One. 2019 Nov 20;14(11):e0225028. doi: 10.1371/journal.pone.0225028 (PMC6867652; doi:10.1371/journal.pone.0225028)
Supplement: S1 Text — (PDF) [file pone.0225028.s002.pdf]

S1 Text. Simulation of participants' investment

To identify the most profitable strategy for the participants, we performed a simulation of both participants and robot confederate investment choices. For each banker and strategy condition, we computed the value of P1 (between 0 and 10). R1 has been calculated by adding or subtracting 3-4 numbers to P1 and all the outcomes exceeding 0 and 10 have been removed. Subsequently, we computed both the values of P2 (between 0 and 10) and R2. R2 has been estimated by following the fixed and collaborative strategy methods reported in the paper. Like for the experiment, every misleading outcome (e.g. a robot collaborative investment resembling a fixed strategy and likewise) has been removed. The simulation included 11909 trials. The final investment (P2+R2) has been tripled and then multiplied by the coefficients used in the experiment. The potential punishment deriving from two different investment choices has also been included. The value obtained has been added to the residual non-invested amount and considered as the payoff. Simulation results are shown in S1 A Fig.

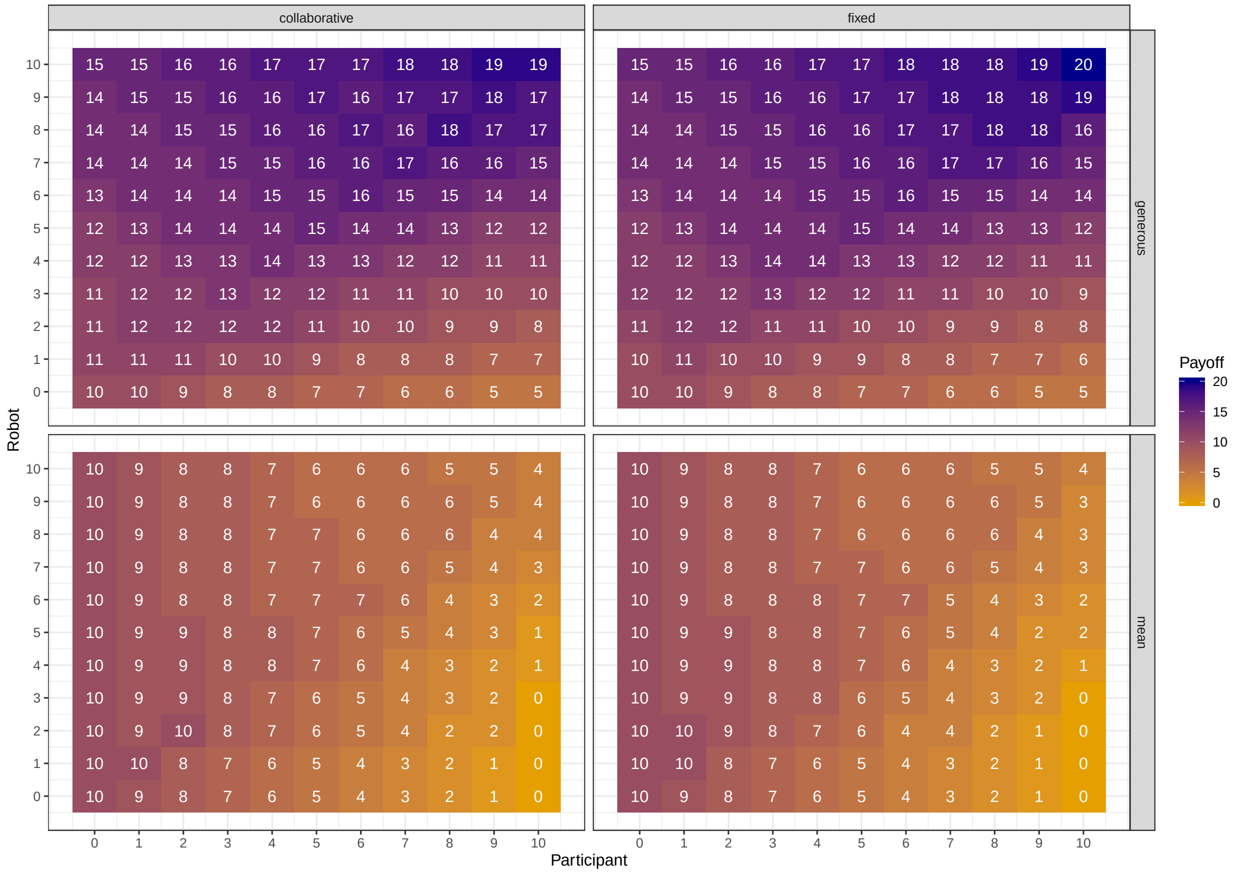

S1 A Fig Simulation results. Average payoff for each combination of both participants' and robot confederate investments are reported for both banker (generous and mean) and strategy (collaborative and fixed) conditions.

In the generous condition, a higher payoff is associated with higher investment. A closer

look at the type of strategy reveals that the participants should be cooperating with the robot confederate to maximise the payoff in the collaborative strategy. In particular, the highest payoff is obtained when both parties invest 10. Moreover, for each of the robot confederate final investment choice, participants' best strategy would be cooperating and investing the same amount. Data from the experiment showed that participants invested on average 6.44 ECU and the robot confederate invested 6.41 ECU. For the fixed strategy, the highest payoff is obtained when both parties invest 10. Differently from the collaborative condition, for each of the robot confederate final investment choice, participants' best strategy would be cooperating or investing more than the robot confederate. Data from the experiment showed that participants invested on average 6.89 ECU and the robot confederate invested 4.90 ECU. Although lower than the simulation results, participants invested overall more than the 50% of their money, pursued a cooperative attitude in the collaborative condition and invested more than the robot in the fixed condition.

In the mean banker condition, a higher payoff is associated with lower investment. A closer look at the type of strategy reveals that for each robot confederate final investment, the best choice for the participants would be investing 0. Moreover, in case the robot confederate invested 2 or 1, the best option for the participants would be cooperating with the robot confederate. Data from the experiment showed that participants invested on average 4.05 ECU and the robot confederate invested 4.12 ECU. For the fixed strategy, the best choice for the participants would be investing 0. In case the robot confederate invested 2 or 1, the best option for the participants would be to invest less than the robot. Data from the experiment showed that participants invested on average 3.74 ECU, where the robot confederate invested 5.33 ECU. Although higher than the simulation, participants invested significantly less than in the generous condition. Moreover, they showed a cooperative attitude in the collaborative condition, while reducing their investment in the fixed condition. Following this evidence, participants' investment pattern seems overall in line with the simulation results.
