# Supplementary material for: Investigating cooperation with robotic peers
Source: PLoS One. 2019 Nov 20;14(11):e0225028. doi: 10.1371/journal.pone.0225028 (PMC6867652; doi:10.1371/journal.pone.0225028)
Supplement: S2 Text — (PDF) [file pone.0225028.s003.pdf]

S2 Text. Preliminary analyses of participants' investment

S2 A Table Participants' initial and final investments mean and SD for both banker conditions (mean/generous) and dependent variable categorisation (cooperative-1/non cooperative-0).

Participants' Initial Investment		Mean (SD)		Mean (SD)
Banker	Generous	7.64 (2.27)	Cooperative category (1)	7.75 (2.48)
			Non Cooperative category (0)	7.55 (2.08)
	Mean	3.91 (2.24)	Cooperative category (1)	4.00 (2.28)
			Non Cooperative category (0)	3.84 (2.20)

Participants' Final Investment		Mean (SD)		Mean (SD)
Banker	Generous	6.63 (2.52)	Cooperative category (1)	6.00 (2.16)
			Non Cooperative category (0)	7.19 (2.68)
	Mean	3.86 (2.47)	Cooperative category (1)	4.67 (2.92)
			Non Cooperative category (0)	3.24 (2.42)

To assess the consistency of the dependent variable, a preliminary analysis of the participants' investment has been performed. For both participants' initial and final investments, data have been compared for the banker condition and have been further grouped on the basis of the dependent variable categories (0 and 1). Moreover, participants' final investment has also been compared to the robot confederate final investment.

Participants' initial investment is shown in S2 B Fig. Investments differed on the basis of the banker condition (t(3248.7) = 47.13, $p < .001$, 95%C.I.$[3.57 - 3.88]$, $d = 1.65$). Participants invested more in the generous banker than in the mean banker, thus initially selecting a correct amount of money to maximise their income (for the generous condition) and minimise the loss (for the mean condition).

For the generous banker, participants' initial investment did not differ between the two categories of the dependent variable (t(1466.8) = -1.76, $p = .079$, 95%C.I.$[-0.43, 0.02]$); regardless their intention to cooperate or not in the second part of the round, participants initially invested a consistent amount of money. Also for the mean banker, participants' initial investment did not differ between the two categories of the dependent variable (t(1530.5) = 1.50, $p = .133$, 95%C.I.$[-0.38, 0.05]$); regardless their intention to cooperate, participants initially invested less than in the generous condition.

Participants' final investment is reported in S2 B Fig. As for their initial investment, participants' final choice differed on the basis of the banker condition (t(32456.7) = 31.68, $p < .001$, 95%C.I.$[2.60, 2.94]$, $d = 1.11$).

For the generous banker, participants' final investment did differ between the two categories of the dependent variable (t(1582.4) = 9.75, $p < .001$, 95%C.I.$[0.94, 1.42]$, $d = 0.48$). Participants' final investment was higher for the trials categorised as non cooperative than

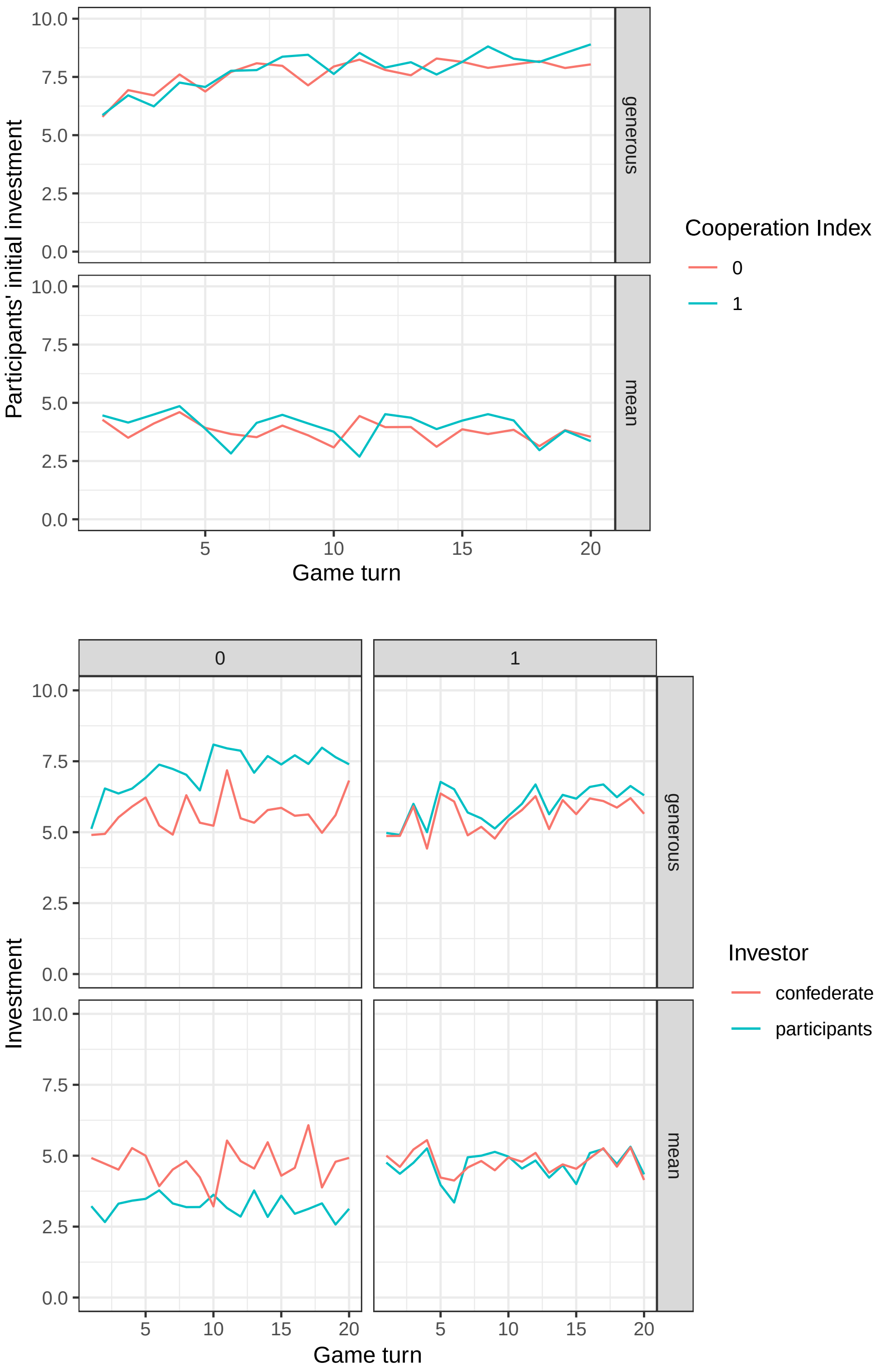

S2 B Fig On the top, participants' initial investment in both banker conditions (mean/generous) and dependent variable categorisation (cooperative-1/non cooperative-0). On the bottom, participants (blue line) and robot (red line) final investments in both banker condition (mean/generous) and dependent variable categorisation (cooperative-1/non cooperative-0).

the cooperative trials. When participants decided to not cooperate, they invested a higher amount of money. Comparisons with the robot confederate final investment, revealed that participants' investment was higher than the robot confederate investment in the non cooperative condition (confederate investment: mean = 5.59, SD = 2.13; t(1635.4) = 13.56, $p < .001$, 95%C.I.$[1.37, 1.83]$, $d = 0.66$). The same result has been found for the cooperative trials (confederate investment: mean = 5.61, SD = 2.13; t(1491.9) = 3.63, $p < .001$, 95%C.I.$[0.17, 0.60]$, $d = 0.18$). However, as the effect size was low, we did not consider this result as reliable.

For the mean banker, participants' final investment did differ between the two categories of the dependent variable (t(1596.3) = -12.98, $p < .001$, 95%C.I.$[-1.66, -1.20]$, $d = 0.60$). Participants' final investment was lower for the trials categorised as non cooperative than the cooperative trials. When participants decided to not cooperate, they invested a lower amount of money. Comparisons with the robot confederate final investment, revealed that participants' investment was lower than the robot confederate investment for the non cooperative condition (confederate investment: mean = 4.65, SD = 2.37; t(1871.3) = -12.72, $p < .001$, 95%C.I.$[-1.62, -1.19]$, $d = 0.59$). No differences have been found for the cooperative trials (confederate investment: mean = 4.76, SD = 2.16; t(1444.7) = -0.79, $p = .429$, 95%C.I. $[-0.32, -0.14]$).

When participants decided to not cooperate, they invested congruently with the type of payoff. That is, they increased their investment for the generous banker to maximise the income and reduced the investment for the mean banker to minimise the loss. When participants decided to cooperate, they invested a similar amount to the robot confederate.
