# Supplementary material for: Investigating cooperation with robotic peers
Source: PLoS One. 2019 Nov 20;14(11):e0225028. doi: 10.1371/journal.pone.0225028 (PMC6867652; doi:10.1371/journal.pone.0225028)
Supplement: S1 Table — (PDF) [file pone.0225028.s004.pdf]

S1 Table. Cronbach's Alpha for each scale.

	**Alpha**
Likeability	0.89
Trust	0.90
Credibility	0.92
Godspeed Questionnaires	
Anthropomorphism	0.89
Animacy	0.87
Likeability	0.90
Intelligence	0.89
Safety	0.27
