# Supplementary material for: Investigating cooperation with robotic peers
Source: PLoS One. 2019 Nov 20;14(11):e0225028. doi: 10.1371/journal.pone.0225028 (PMC6867652; doi:10.1371/journal.pone.0225028)
Supplement: S2 Table — (PDF) [file pone.0225028.s005.pdf]

S2 Table. List of mean and standard deviation values for all the scales in each sub-condition of banker, behaviour and strategy.

	Banker				Behaviour				Strategy			
	Generous		Mean		Anthropomorphic		Mute		Collaborative		Fixed	
	mean	SD	mean	SD	mean	SD	mean	SD	mean	SD	mean	SD
Likeability	4.21	0.93	4.05	0.90	4.27	0.99	3.99	0.82	4.15	0.99	4.12	0.84
Trust	4.01	1.13	4.22	1.17	4.51	1.11	3.72	1.05	4.21	1.07	4.02	1.23
Credibility	4.85	0.76	4.93	0.73	5.08	0.72	4.71	0.73	4.98	0.71	4.82	0.79
Anthrpomorphism	2.49	0.79	2.45	0.77	2.39	0.72	2.55	0.84	2.47	0.83	2.47	0.73
Animacy	2.72	0.48	2.82	0.68	2.95	0.56	2.59	0.61	2.82	0.58	2.72	0.59
Likeability	3.83	0.59	3.71	0.68	3.99	0.62	3.54	0.58	3.77	0.61	3.77	0.67
Intelligence	3.78	0.57	3.85	0.51	3.87	0.56	3.76	0.52	3.88	0.54	3.75	0.53
Safety	4.02	0.44	4.10	0.40	4.08	0.37	4.04	0.48	4.05	0.34	4.07	0.50
