# Supplementary material for: Investigating cooperation with robotic peers
Source: PLoS One. 2019 Nov 20;14(11):e0225028. doi: 10.1371/journal.pone.0225028 (PMC6867652; doi:10.1371/journal.pone.0225028)
Supplement: S1 File — (PDF) [file pone.0225028.s006.pdf]

S1 File. Copy of the adapted questionnaires used in the study.

You are kindly asked to fill in four short questionnaires.
Reflect on the experience you had interacting with the robot during the previous sessions, and give numerical ratings using the different scales provided in each questionnaire.
For each question, circle the number that best reflects your rating.

Q1 – Questionnaire for Likeability Measurement.
(From Rau et al. Computers in Human Behavior, 2009)

	Strongly disagree						Strongly agree
This robot is friendly	1	2	3	4	5	6	7
This robot is likeable	1	2	3	4	5	6	7
This robot is warm	1	2	3	4	5	6	7
This robot is approachable	1	2	3	4	5	6	7
I would ask this robot for advice	1	2	3	4	5	6	7
I would like this robot as a co-worker	1	2	3	4	5	6	7
I would like to be friends with this robot	1	2	3	4	5	6	7
This robot is physically attractive	1	2	3	4	5	6	7
This robot is similar to me	1	2	3	4	5	6	7
This robot is knowledgeable	1	2	3	4	5	6	7

Q2 – Questionnaire for Trust Measurement.
(From Rau et al. Computers in Human Behavior, 2009)

	Strongly disagree						Strongly agree
This robot was sincere	1	2	3	4	5	6	7
This robot was interested in talking with me	1	2	3	4	5	6	7
The robot wanted me to trust him/her	1	2	3	4	5	6	7
The robot was willing to listen to me	1	2	3	4	5	6	7
The robot was open to my ideas	1	2	3	4	5	6	7
The robot was honest in communicating with me	1	2	3	4	5	6	7

S1 File. Copy of the adapted questionnaires used in the study.

Q3 – Questionnaire for Source Credibility Measurement.

(From Rau et al. Computers in Human Behavior, 2009)

Please rate your impression of the robot on these scales:

Unintelligent	1	2	3	4	5	6	7	Intelligent
Incompetent	1	2	3	4	5	6	7	Competent
Dishonest	1	2	3	4	5	6	7	Honest
Sinful	1	2	3	4	5	6	7	Virtuous
Selfish	1	2	3	4	5	6	7	Unselfish
Low character	1	2	3	4	5	6	7	High character
Inexpert	1	2	3	4	5	6	7	Expert
Stupid	1	2	3	4	5	6	7	Bright
Untrained	1	2	3	4	5	6	7	Trained
Uninformed	1	2	3	4	5	6	7	Informed
Unsympathetic	1	2	3	4	5	6	7	Sympathetic
Untrustworthy	1	2	3	4	5	6	7	Trustworthy

Q4 – Godspeed Questionnaires.

(From Bartnek et al. International Journal of Social Robotics, 2009)

ANTROPOMORPHISM

Please rate your impression of the robot on these scales:

Fake	1	2	3	4	5	Natural
Machinelike	1	2	3	4	5	Humanlike
Unconscious	1	2	3	4	5	Conscious
Artificial	1	2	3	4	5	Lifelike
Moving Rigidly	1	2	3	4	5	Moving elegantly

S1 File. Copy of the adapted questionnaires used in the study.

ANIMACY

Please rate your impression of the robot on these scales:

Dead	1	2	3	4	5	Alive
Stagnant	1	2	3	4	5	Lively
Mechanical	1	2	3	4	5	Organic
Artificial	1	2	3	4	5	Lifelike
Inert	1	2	3	4	5	Interactive
Apathetic	1	2	3	4	5	Responsive

LIKEABILITY

Please rate your impression of the robot on these scales:

Dislike	1	2	3	4	5	Like
Unfriendly	1	2	3	4	5	Friendly
Unkind	1	2	3	4	5	Kind
Unpleasant	1	2	3	4	5	Pleasant
Awful	1	2	3	4	5	Nice

PERCEIVED INTELLIGENCE

Please rate your impression of the robot on these scales:

Incompetent	1	2	3	4	5	Competent
Ignorant	1	2	3	4	5	Knowledgeable
Irresponsible	1	2	3	4	5	Responsible
Unintelligent	1	2	3	4	5	Intelligent
Foolish	1	2	3	4	5	Sensible

PERCEIVED SAFETY

Please rate your emotional state on these scales:

Anxious	1	2	3	4	5	Relaxed
Agitated	1	2	3	4	5	Calm
Quiescent	1	2	3	4	5	Surprised

3
